# Supporting migrants and refugees with posttraumatic stress disorder: development, pilot implementation, and pilot evaluation of a continuing interprofessional education for healthcare providers

**DOI:** 10.1186/s12909-020-02220-3

**Published:** 2020-09-16

**Authors:** Stefan Jobst, Matthias Windeisen, Alexander Wuensch, Michael Meng, Christiane Kugler

**Affiliations:** 1 Faculty of Medicine, Institute for Nursing Science, University of Freiburg, Breisacher Str. 153, 79110 Freiburg, Germany; 2 Department of Psychosomatic Medicine and Psychotherapy, Freiburg University Medical Center, Hauptstraße 8, 79104 Freiburg, Germany

**Keywords:** Posttraumatic stress disorder, Migration, Refugee, Interprofessional continuing education, Healthcare, Pilot study, Kirkpatrick model, Satisfaction, Knowledge

## Abstract

**Background:**

Refugees and migrants face an increased risk of developing posttraumatic stress disorder (PTSD). Adequate care can be insufficient due to language barriers, cultural differences, and knowledge deficits of health service providers. Therefore, professional associations requested that healthcare providers to be educated to provide culturally sensitive care. An evidence-based educational intervention in the form of a continuing interprofessional education (CIPE) for healthcare providers on the topic of PTSD in migrants and refugees was developed, pilot-implemented, and evaluated according to the first two levels of the Kirkpatrick evaluation model (reaction and learning).

**Methods:**

The development of a curriculum for the CIPE intervention was based on a narrative literature review. Its content was validated by experts (*N* = 17) in an online survey and analyzed using both the Content Validity Index and a thematic analysis. The evaluation of the CIPE intervention was performed by conducting a pilot study with a quasi-experimental single group, using a pre-posttest design. In total, there were 39 participants distributed among three pilot courses. We collected and analyzed data on satisfaction, knowledge, and feasibility.

**Results:**

The curriculum for a half-day course, consisting of 8 modules, showed almost excellent content validity (S-CVI = 0.92). In the pilot-implementation phase, participants were “very satisfied” with the pilot courses and a positive effect on their knowledge was detected. No correlation between satisfaction and knowledge gain was found.

**Conclusions:**

The CIPE intervention can be considered feasible and seems promising in its effects on satisfaction and knowledge. The insights gained in this study can be used to adapt and optimize the educational intervention, whereby the feedback from course attendees is particularly useful. Future studies need to further examine the effects in larger samples and more robust study designs.

## Background

Worldwide, 258 million migrants were accounted for in 2017 [1]. Of these, 70.8 million people were refugees forcibly displaced internally (41.3 million), internationally (25.9 million), or asylum seekers (3.5 million) in order to escape unbearable or life-threatening situations [2]. In Germany, more than 1.8 million asylum applications were submitted between 2014 and 2018 [3] with the majority of people wanting to escape war, persecution, unstable living conditions, or human rights violations [4].

Migrants and refugees both, are at increased risk to suffer from common mental disorders like depression, posttraumatic stress disorder (PTSD), and/or anxiety, as compared to the general population [5]. Exposure to stressors and traumatic events before, during, and after migration has been identified as a major associated risk factor [6–8].

PTSD is a psychiatric disorder that is a potential consequence of one or more traumatic events experienced by oneself or by someone else that result in feelings of helplessness and a shattered self-concept and view of the world [9, 10]. A traumatic event is characterized by the discrepancy between the subjectively experienced threat and pre-existing coping strategies of the individual [10]. The frequency of PTSD depends on the type of trauma [11] and protective and risk factors present [12]. Core symptoms of PTSD are intrusive thoughts, hyperarousal, and avoidance behavior [9]. The international prevalence of PTSD is 1–2% in the general population and ranges between 9 and 36% in refugees [5], and 4–86% in long-term war-refugees [13]. This elucidates that refugees can be seen as a “highly vulnerable” group [14], having a ten-fold higher risk for PTSD [15]. A systematic review focusing on the period between 1990 and 2014 in Germany, reported PTSD prevalence rates in migrants or refugees ranging from 16 to 55% depending on sample sizes, sampling methods and assessment instruments used [16]. In addition, higher PTSD rates were observed not only among newly arrived refugees or migrants, but also among migrants with an average stay of more than 10 years in the host country [17].

Access to health care is often limited to acute disease, pain, and life threatening conditions and does not always include psychotherapy [18, 19]. In addition, differences in cultural beliefs and expectations, lack of trustworthiness towards health services, and language barriers [18–20] may lead to inadequate access to and provision of healthcare of (mentally) ill and/or traumatized migrants and refugees [20, 21].

In contrast, evidence from reviews suggests that utilization of somatic healthcare services in Europe by this group of individuals is higher compared to non-migrants [22, 23] and that the length of stay in hospitals tend to be longer [23]. As PTSD is associated with somatization [24], and those affected more often complain of pain or physical symptoms without reference to traumatic events [25, 26], it can be concluded, albeit with caution, that migrants and refugees may likely present at primary medical care settings with PTSD symptoms masked by somatic symptoms [26].

However, primary healthcare professionals (e.g. physicians, nurses, social workers) may feel inadequately prepared to care for traumatized patients [27, 28], and reveal deficits in knowledge and education regarding PTSD as compared to mental health service providers [29, 30]. This may result in PTSD being unrecognized [29] and patient reactions may not be interpreted within the context of PTSD. Furthermore, primary healthcare professionals often report differences in cultural understanding of health and healthcare in refugees as challenging [28].

To this point, the German Association for Psychiatry, Psychotherapy and Psychosomatics [31] and the German National Academy of Sciences Leopoldina [32], both request that primary healthcare providers – especially those not solely practicing in psychotherapeutic settings – should be educated concerning potential health problems and psychological symptoms of mental disorders like PTSD in the migrant and refugee population. Moreover, the awareness of primary healthcare providers should be raised for the psychosocial situation of migrants and refugees to improve cultural-sensitive care. This is in line with the recommendation of the International Council of Nurses to include “health issues associated with population movement, including culture and gender sensitivity training and the unique needs of migrants and refugees” (p.3) in continuing education [33]. Nevertheless, many primary healthcare professionals report a lack of continuing education in this field and subsequently urge for more training, education and/or guidance for the care of such patients, in order to improve their practice [28]. As this includes different healthcare professions, an interprofessional approach in continuing education is required to meet above-mentioned demands [34].

A commonly used framework for the evaluation of continuing educational programs is the four levels provided by Kirkpatrick & Kirkpatrick [35]. Level 1 (Reaction) refers to participants’ satisfaction with respect to the educational programs. Level 2 (Learning) implies participants’ knowledge acquisition including changes in attitude or increase of skills. Level 3 (Behavior) refers to participants’ behavior changes as a result of completing the program. Level 4 (Results) refers to the end result and effect of the educational program.

As PTSD is a highly prevalent health burden among migrants and refugees, and given that barriers - both on the part of those affected and on the part of healthcare service providers may lead to inadequate care - training and continuing education for healthcare professionals could positively contribute to an improvement of care in host countries such as Germany [31]. Therefore, the purpose of this study was to develop an evidence-based CIPE intervention on “posttraumatic stress disorder symptoms in patients with flight and migration history” for healthcare professionals in acute care settings. Furthermore, we aimed to implement and evaluate this CIPE intervention within three pilot courses with respect to healthcare professionals’ satisfaction with, and change of knowledge concerning the contents of the CIPE intervention.

## Methods

Reporting on this pilot trial was guided by the recommendations of the revised Standards of Quality Improvement Reporting Excellence (SQUIRE 2.0) publication guidelines [36]. The description of the CIPE and the pilot courses was guided by the recommendations of the Guideline for Reporting Evidence-based practice Educational interventions and Teaching (GREET) [37].

### Design and setting

**Fig. 1:**
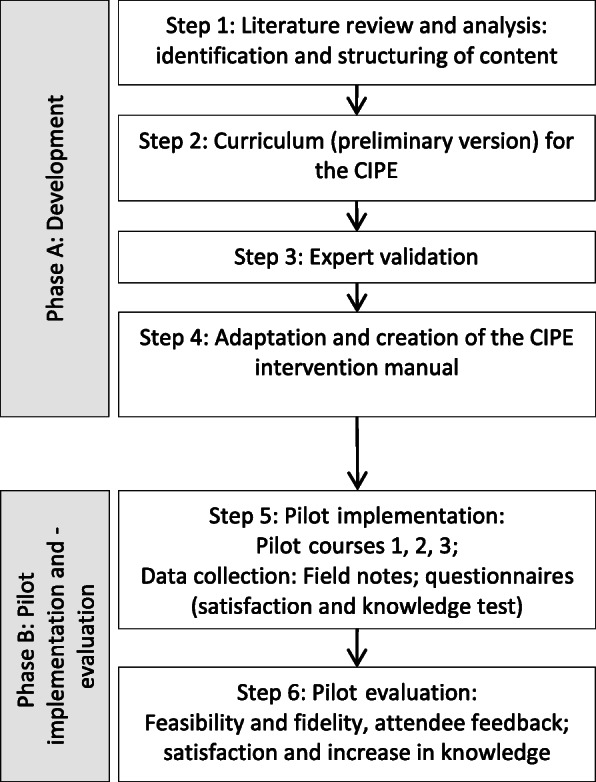
Phases and steps of development and evaluation of the educational intervention. CIPE = Continuing interprofessional education

This pilot study was designed as a quasi-experimental single group, pre-posttest study at two university medical centers in Germany. Phases and steps of development and evaluation of the CIPE intervention are shown in Fig. 1. The Pilot study followed the conditions of the Declaration of Helsinki and obtained approval from the Ethics Committee at the University of Freiburg (Registration number: 479/18) and the staff councils of the two university medical centers.

### Development of the CIPE intervention (phase A)

An interprofessional project group consisting of nurses with expertise in continuing education and psychiatric nursing, and a psychotherapist and medical educator developed the curriculum for the CIPE intervention addressing the requirements of being evidence-based, practical, sustainable, and broadly applicable.

#### Literature review and structuring of content (step 1)

The content for the CIPE was identified by a narrative literature review [38] of international literature via bibliographic databases and academic search engines using keywords encompassing the umbrella terms “posttraumatic stress disorder”, “flight”, “migration”, and “healthcare”. Relevant literature was analyzed and its content was assigned to different overarching themes, which were reviewed by the members of the interprofessional project group (SJ, MW, AW, MM, CK) and adapted, if necessary. Finally, themes were structured and condensed into major subject areas for using them as a basis for the development of the curriculum.

#### Preliminary curriculum (step 2)

Contents from these major subject areas were combined into teaching units, so-called modules, taking into consideration the principles of adult learning within the context of the constructivist learning theory [39, 40]. These modules were listed in tabular form and served as curriculum with additional information on the respective duration, learning objectives, teaching methods, and references for the respective module. This preliminary version of the curriculum formed the basis for the validation by a panel of experts.

#### Content validation by an expert panel (step 3)

Experts for content validation were defined according to Polit & Beck [41] as “people with strong credentials” with respect to key issues of the CIPE intervention that are “knowledgeable about the target population” (p. 336). In a purposive sampling approach, experts were identified through authorship of key publications concerning the topics of the CIPE intervention, national organizations, internet searches, and recommendations of members of the project group. Inclusion criteria for national experts were: (1) a proven expertise in at least one of the following key criteria: PTSD, education, nursing and/or medicine, and (2) speaking and understanding the German language. Eligible experts were contacted via e-mail. The email provided information regarding the project and asked whether they would be willing to participate in an expert panel to evaluate the newly developed curriculum draft. If written informed consent was obtained, a link to an online survey (EvaSys [42]) designed based on the preliminary draft version of the curriculum was sent to the expert. In this survey, the modules could be rated concerning (a) the relevance of the content, (b) the suitability of educational methods, and (c) the practical relevance on 4-point Likert-scales ranging from ‘very relevant/suitable’ (=1) to ‘not relevant/suitable’ (=4) or (d) designated as ‘I cannot judge this item’. Additionally, at the end of the survey, experts had the opportunity to comment on individual items and on the curriculum as a whole, and could voluntarily provide information on their expertise. Data from the survey were analyzed by calculating the Content Validity Index (CVI) on item- (I-CVI) and scale-level (S-CVI). These indices allow for an assessment of the content validity based on the aggregation of expert ratings [41] and gather the extent to which the individual items (modules) represent the construct to be determined [43]. According to Polit & Beck [41], content validity is considered to be excellent when all I-CVIs are higher than 0.78 and the S-CVI is at least 0.90.

#### Adaptation and creation of the CIPE intervention manual (step 4)

Results of the expert validation formed the basis for curriculum modification, as determined through discussion and reflections in project group sessions. The adapted version of the curriculum served as the foundation for the CIPE intervention manual. The manual contained a more detailed elaboration of the CIPE content and its implementation.

### Pilot implementation and evaluation (phase B)

#### CIPE pilot courses (step 5)

Originally, two pilot courses were planned and promoted via flyers, a web page, and intranet advertisements. The target audience initially consisted of healthcare providers of adult somatic acute care settings, social workers, medical and nursing students, and nurses in vocational training. The focus on personnel of acute adult healthcare settings was justified by the fact that, compared to adults, symptom representation and therapy of PTSD differs in children and adolescents depending on developmental stage [44, 45]. Furthermore, we assumed that professionals working in psychiatric settings would already have sufficient knowledge and skills related to PTSD.

Due to numerous requests during the registration process from individuals not matching the original target audience, we decided to allow these individuals to attend during the pilot phase. Thus, individuals working in pediatric or psychiatric settings, or who volunteer or work in educational settings with migrants and refugees, also attended the pilot courses. As a result of the vast interest in the CIPE, we planned and promoted a third course. This pilot course was opened to people currently or potentially professionally involved in the care of migrants or refugees.

Attendance in the pilot courses was free of charge. Attendees affiliated with one of the university medical centers were able to count their attendance as working time. All attendees were able to claim continuing education credit points for their respective profession from the German organization ‘Registration of Professional Nurses’ or the ‘State Medical Chamber’.

All CIPE pilot courses involved the same two instructors and a teaching assistant. The first instructor (SJ) is a nurse scientist at master level with university-based teaching experience. The second instructor (AW) is a psychotherapist at PhD level and medical educator. The teaching assistant (MW) is a registered mental health nurse at bachelor level and experienced in the care of traumatized patients.

#### Pilot evaluation of CIPE courses (step 6)

The pilot courses were evaluated concerning feasibility of the CIPE intervention and fidelity of its delivery and with respect to the first two levels according to the framework by Kirkpatrick & Kirkpatrick [35], i.e. reaction (level 1) and learning (level 2).

Participants for the pilot study were recruited from pilot course attendees. Participants were included if they (1) were at least 18 years old, and (2) had sufficient CIPE program language knowledge (German). Exclusion criterion was incomplete participation (e.g. early leave) from the CIPE pilot courses. Participation in the pilot study was not a prerequisite for attendance in the pilot courses. All attendees were written and verbally informed at the beginning of each pilot course regarding the evaluative character of the educational sessions. Upon receiving signed informed consents, participants received the knowledge test and questionnaires. The pilot course began after knowledge test completion. At the end of the course, participants received the identical knowledge test followed by the ABC-SAT questionnaire and questions about occupation, professional qualification, age, gender, and previous experience regarding the content of the CIPE intervention.

### Measures

To gain feasibility and fidelity information on the CIPE intervention, each pilot course was observed by a project group member. Information regarding course sequence, content delivery time, specific peculiarities and attendee feedback (devoid of personal data) was noted.

To assess reaction, we used the ABC-SAT questionnaire, an instrument developed to measure satisfaction with continuing education. This questionnaire was administered after the intervention and consists of 11 items, divided into 3 sub-scales (affective, behavioral, cognitive), each of which is graded on a 5-point scale. Scores can range from 0 (absolute dissatisfaction) to 44 (highest satisfaction) points. During the development phase of the ABC-SAT questionnaire an expert panel of health-care professionals (nurses and physicians) attested face validity of the instrument. Construct validity was tested with a principal component analysis and internal consistency (Cronbach’s Alpha) was tested being α = .60 for the affective subscale, α = .66 for the behavior subscale, and α = .83 for the cognitive subscale [46].

Learning was assessed by a self-developed written knowledge test (see Additional file 1) that was administered before and immediately after the CIPE intervention. The test consisted of 9 multiple choice questions regarding key topics of the CIPE intervention, each with 4 possible answers and 18 correct answers. Correct and incorrect answers were counted on item-level. The change in knowledge was quantified by the difference of scores on item-level between pre- and post-assessments with a possible range from − 18 to 18.

### Analysis

#### Content validation using the CVI by the expert panel (step 3)

We calculated the I-CVI by dividing the number of experts who rated the respective item as “1” or “2” by the total number of experts who rated that item. The S-CVI was calculated by computing the mean of the I-CVIs. Using an inductive approach, expert comments and field notes were analyzed thematically.

#### Pilot evaluation of CIPE courses (step 6)

Demographic data and data on satisfaction and knowledge of the participants of the pilot courses were analyzed using descriptive statistics. Data distribution was tested using the Shapiro-Wilk-Test. Parametric tests were calculated for normally distributed variables, and non-parametric tests for not normally distributed variables. Additionally, the change in knowledge was analyzed by calculating a Wilcoxon signed-rank test. To identify possible correlations between satisfaction and learning, a Kendall’s Tau rank correlation was calculated. Cases with missing data were excluded from the calculation of correlations. Statistical significance was set a priori at *p* ≤ 0.05. Calculations were performed using IBM SPSS Statistics Version 22.

## Results

### Development of the CIPE intervention (phase A)

#### Development of the preliminary version of the curriculum for the CIPE (steps 1 and 2)

The content for the curriculum was extracted from different publication formats identified by the literature review. The resulting preliminary version of the CIPE curriculum consisted of eight modules with a total duration of 4.5 h. Modules 1 and 8 were formal modules providing general information for attendees before beginning and upon finishing the CIPE. Relevant course content was condensed in modules 2 to 6. The main teaching methods considered were lectures, with the exception of modules 2 and 5, in which group work and an exercise (moderated role-play) were to take place, respectively.

#### Expert validation of the preliminary version of the curriculum for the CIPE (step 3)

We identified 52 potential experts of which 21 consented to participate in the survey (recruitment rate = 40.4%). Of these, 17 experts completed the survey (response rate = 80.9%). Characteristics of these experts are displayed in Table 1.

**Table 1:** Characteristics of experts (*n* = 17) for content validation (step 3)

	n (%)
Expertise^a^	
nursing practice	8 (47.1)
medical practice	4 (23.5)
psychotherapy	5 (29.4)
psychosocial practice	5 (29.4)
education/pedagogy/didactics	7 (41.2)
others	5 (29.4)
Professional experience with PTSD^a^	
theoretical experience	10 (58.8)
practical experience	11 (64.7)
none^b^	1 (5.9)
Professional experience with migration/flight^a^	
theoretical experience	8 (47.1)
practical experience	13 (76.5)
none	2 (11.8)
Academic qualification level	
Master	8 (47.1)
Diploma	2 (11.8)
Doctorate	6 (35.3)
none	1 (5.9)

^a^ = multiple responses possible; PTSD = Posttraumatic stress disorder; ^b^ = proven expertise in PTSD not essential for participation in expert rounds (see inclusion criteria)

Calculated I-CVIs for the relevance of the content, the suitability of teaching methods, and the practical relevance of the modules are shown in Table 2. With the exception of the I-CVI on the practical relevance of module 4, all other I-CVIs were above the recommended threshold of 0.78 [41]. The S-CVI was 0.92. Expert comments were summarized and key statements obtained through a thematic analysis. Despite a good I-CVI rating as a teaching method, some experts were critical of using a role-play in this CIPE (module 5). They argued that group dynamics over the short duration of this CIPE may not be fitting to apply this method effectively and could possibly result in an overextension of attendees. With respect to module 4, two experts commented on the limitation of the practical relevance with the lack of a connection to everyday clinical practice with too much of a strong focus on classical PTSD symptoms.

**Table 2:** Module I-CVIs of the preliminary version of the curriculum for the CIPE intervention with initial titles (step 3)

I-CVI	Modules (initial titles)							
	Formal introduction	Thematic introduction	Background information	Handling symptoms	Exercise (Role-play)	Outlook	Summary	Feedback/ Farewell
	1	2	3	4	5	6	7	8
relevance of the content	−//−	1.00	1.00	1.00	0.94	1.00	1.00	−//−
suitability of teaching method	−//−	0.94	0.88	0.80	0.94	0.82	0.94	−//−
practical relevance	−//−	1.00	0.82	0.75	0.94	0.94	0.92	−//−

*I-CVI*   Content Validity Index on item level

#### Revision of the curriculum after expert validation (step 4)

Due to the high I-CVI ratings of experts, the structure of the curriculum as proposed in the preliminary version (eight modules plus respective key subjects) was maintained. The following adaptations were necessary after taking into account the CVI ratings and the comments of the expert panel.

The content in all modules has been shortened or partly deleted and an additional break was scheduled (between modules 5 and 6). The time quotas have been slightly increased (modules 3 and 4) or slightly reduced (module 5), and made more flexible overall. We implemented more interactive teaching and learning methods such as discussions and/or exchange of experiences (modules 2, 5, 7), and a self-directed learning approach in the form of group work instead of role-play (module 5). The content in module 4 was linked to many examples from everyday clinical practice. Furthermore, we included three video clips to enhance the learning experience of lectures and to involve the perspective of affected individuals. In order to accomplish the latter, we on the one hand, used publicly available video-clips with reports of traumatized refugees, and on the other hand videotaped an interview with a PTSD affected individual regarding personal experiences. Finally, after revisions were made, we created a CIPE intervention manual.

### Description of the CIPE intervention

Direct learning objectives of the CIPE are: (1) the sensitization and increased awareness of attendees on the topic of PTSD within the context of flight and migration; and (2) the provision of knowledge and skills for the culturally-sensitive care of people with such experiences and having symptoms of PTSD. Overall indirect learning objectives are: (1) the improvement of interprofessional communication; and (2) the improvement of care for people with flight and migration experiences and symptoms of PTSD.

The CIPE is a standardized, single-session, face-to-face educational intervention for groups containing 7–24 attendees (with a focus on professionals within healthcare settings), two instructors and a duration of 4.5–5.5 h. Variability in the duration of the CIPE is dependent upon the number of attendees. Two separate rooms are required to provide space for small group work. Instructors require pedagogic-didactic competencies and knowledge on the topics of PTSD, migration and healthcare.

An overview of the final schedule for the eight consecutive modules of the CIPE intervention is displayed in Table 3 (for a detailed description of the content see Additional file 2). Exchange-based (interactive), and received learning (didactic) approaches are used to deliver the content [47], while employing multimedia and various teaching formats to serve diverse learning styles. Modules 1 and 8 provide the course framework, while actual course content is delivered in modules 2 through 7. In addition to the course itself, attendees receive a pocket card specifically developed for this CIPE, based on published literature and summarizing key points of module 4 (for a detailed description see Additional file 2). Furthermore, attendees receive another document with additional information and resources. During the two breaks, attendees have the opportunity to informally exchange views and experiences with each other.

**Table 3:** Final version structure and content overview of the CIPE curriculum intervention (step 4)

Module	Topic(s)	Educational method	Duration (minutes)
1	Welcome/Formal introduction	–	10
2	Thematic introduction	discussion, experience exchange	20–30
3	Background information on trauma, PTSD & flight/migration	lectures, video	50–60
–	Break 1	–	30
4	Handling symptoms of PTSD	lecture, brochure (pocket card), video	45–60
5	Interprofessional handling of symptoms of PTSD in refugees/migrants	self-directed learning, group work, experience exchange, presentation	45
–	Break 2	–	15–20
6	Outlook on further aspects of healthcare of refugees/migrants	lecture	30
7	Summary of key issues of the CIPE	experience exchange, discussion; handout	15–20
8	Feedback and Farewell	–	15–20

*CIPE*   Continuing interprofessional education, *PTSD*   Posttraumatic stress disorder

### Pilot implementation (phase B; step 5)

**Fig. 2:**
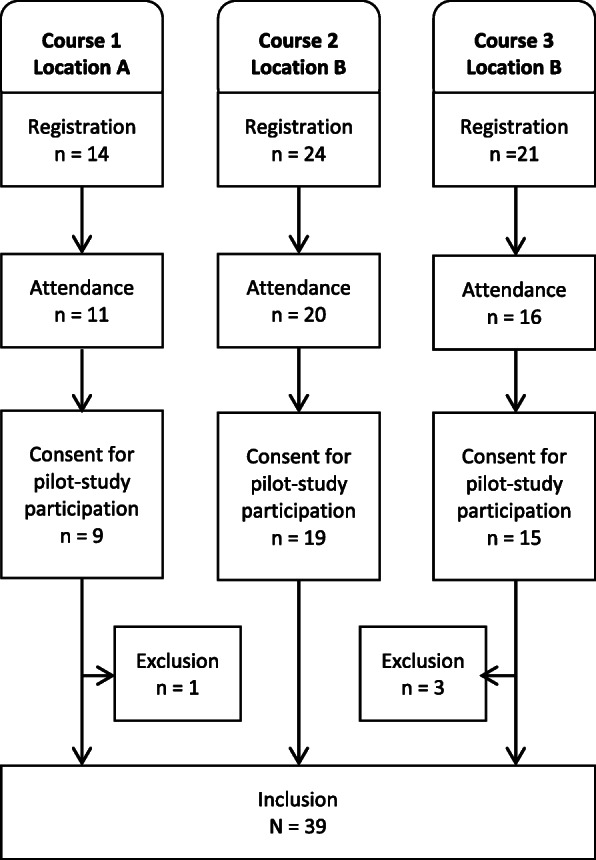
Flowchart of registration for the pilot courses, course attendance, and pilot study participation

Three CIPE pilot courses were conducted at two university-based medical centers in two different German areas between January and March 2019. A maximum of 72 individuals (24 per course) could take part in the pilot courses. Of 59 registered individuals, 47 ultimately attended, equating an attendance rate of 65.3%. Figure 2 shows detailed information on registration and attendance for the individual courses.

All content of the documents, the course methods, and procedures were standardized. Attendees were also given additional relevant PTSD-specific information for their regions.

### Pilot evaluation (phase B; step 6)

#### Field notes and attendee feedback

Field notes confirmed that all three pilot courses were conducted as designed and the content of all modules were delivered in the estimated time frame. Due to attendee requests during the first course for information regarding psychotherapy, we modified the content of module 3 to include psychotherapy fundamentals. Attendees’ feedback upon course completion positively emphasized good course organization, clear and structured content, use of different media and teaching methods, and the interprofessional learning experience. Furthermore, attendees made suggestions for additional course improvement with respect to teaching methods (e.g. integration of attendees’ own practice examples), content (e.g. outlook on those affected without a migratory background), and organization (e.g. more time for [informal] interprofessional exchange).

#### Results of the pilot study

Thirty-nine individuals with a mean age of 44.2 years (range 22–62; SD 12.3) took part in the pilot study to evaluate the CIPE pilot course (recruitment rate = 83.0%) (Fig. 2). Most of the participants (*n* = 22) were between 40 and 59 years old. Their characteristics are displayed in Table 4. The majority of the participants was female working in the nursing profession. One third of the participants belonged to other professions. Slightly less than half of the participants had an academic degree. Most of participants had previous experiences with PTSD and/or in the care of migrants/refugees. We then created three broad categories to summarize and describe participants based on their heterogeneous background (participants could have been allocated to more than one category): Almost 18% worked in a psychiatric setting, 74% had direct patient contact in their work, and 10% were active in the field of education.

**Table 4:** Characteristics of participants (*N* = 39) of the pilot study for the evaluation of the CIPE pilot courses (step 6)

	n (%)
Gender	
female	30 (76.9)
male	9 (23.1)
Migration background	
yes	8 (20.5)
no	31 (79.5)
Professional education/Profession^a^	
Physician	4 (8.9)
Nurse	20 (44.4)
Medical Student	3 (6.7)
Nursing Student	3 (6.7)
Other	15 (33.3)
Academic degree	
Bachelor	2 (5.1)
Master	8 (20.5)
Diploma	2 (5.1)
Doctorate	6 (15.4)
None	21 (53.9)
Previous experiences in the care of PTSD	
yes	28 (71.8)
no	11 (28.2)
Previous experiences in the care of migrants/refugees	
yes	30 (76.9)
no	9 (23.1)

^a^ = Due to multiple statements the total number of statements was *n* = 45, *CIPE*   Continuing interprofessional education, *PTSD*   Posttraumatic stress disorder

#### Satisfaction

Thirty-nine participants completed the ABC-SAT questionnaire. The overall mean score was 38.9 points (SD = 3.6) ranging from 30 to 44 points (see Table 5).

**Table 5:** Rating scores of the sub-scales of the ABC-SAT questionnaire (*n* = 39)

Sub-scale	Range of possible points	Mean (SD; Range)
Affective	0–12	10.9 (0.97; 9–12)
Behavioral	0–8	7.6 (0.13; 5–8)
Cognitive	0–24	20.4 (0.41; 14–24)

*SD*   Standard deviation, higher ratings indicate more satisfaction

#### Change in knowledge

Twenty-five participants (64.1%) answered more questions correctly in the post-test than before the intervention. The median increase in correctly answered questions was 2. Another 10 participants (26.6%) did not differ in correctly answered questions before and after the CIPE intervention. Based on all answers of all participants, 79.1% of the pre-test and 86.3% of the post-test answers were correct. This represents an increase of 7.2% from pre- to post-test. Table 6 depicts the results of the knowledge test, before and after the CIPE intervention. Both the high median number of correct answers and the low median number of incorrect answers were in the opposite margin areas of possible values, respectively. The comparison of median scores pre- and post-evaluations showed that, with a statistically significant increase in the number of correctly answered questions and the difference between correct and incorrect answers, more questions were answered correctly after the intervention. The median number of incorrectly answered questions remained the same.

**Table 6:** Comparison of the number of correct and incorrect answers in the knowledge test before and after the CIPE intervention (step 6)

Variable (possible range)	pre-intervention	post-intervention	significance test
	Median (IQR)		
Correct answers (0–18)	14 (3)	16 (1)	≤ .003^a^
Incorrect answers (0–18)	4 (3)	4 (2)	.117^a^
Difference of correct and incorrect answers (−18–18)	10 (5)	12 (3)	≤ .003^a^

^a^ = Wilcoxon signed-rank test (Bonferroni corrected), *IQR*   Interquartile range

We did not find any correlation between satisfaction and change in knowledge (*τ* = .111; *p* = .364).

### Missing data

The rate of missing data in the expert validation was 0.84%. With respect to participants’ demographic data, 7.7% did not state any subject area or department of professional activity, and 2.6% made an invalid entry. There were no missing data in the ABC-SAT questionnaire and the knowledge test.

## Discussion

In this article, we reported about the development, pilot implementation and pilot evaluation of a CIPE intervention concerning healthcare for migrants and refugees with PTSD. The development of the curriculum for the CIPE was based on international literature and the principles of adult learning and interprofessional education. Its content was validated by experts and was successfully implemented in a pilot phase where participants of the pilot courses showed high satisfaction with this CIPE and a knowledge gain.

Content validation of the preliminary version of the curriculum for the CIPE was performed by experts covering a wide range of expertise with different levels of qualification. With the exception of one, all I-CVIs were rated above the threshold of 0.78 resulting in a S-CVI of 0.92. This implies nearly excellent overall content validity [41]. A possible explanation for the lower I-CVI rating of the practical relevance in module 4 could be that we did not provide a detailed description of the content of the pocket card in the preliminary version of the curriculum. However, the pocket card was explicitly designed for practical application and therefore provides advice and guidance for situations practitioners face in daily work. The comments on this module also revealed that the content recommended by the experts to enhance practical relevance was largely the same as that on the pocket card. In general, the I-CVI values, in combination with the analysis of the expert comments, enabled us to make targeted changes to the curriculum in order to optimize it. Although two rounds of expert review are recommended [41], we only undertook one. However, a second round did not seem necessary. This is because of the number of participating experts, which was significantly higher than recommended (8–12 experts [41]), and because of the good content validity, suggesting that major and/or fundamental changes to the curriculum were not needed.

The overall attendance in pilot courses was 65.3%. On the one hand, with the exception of the second course, the maximum number of possible registrations was not met; on the other hand, one fifth of the interested individuals cancelled their registration shortly before the course dates or did not show up. Potential barriers could have been staff shortages, time constraints, limited interest in the topic, or concerns about interprofessional education. Furthermore, marketing and/or information dissemination limitations could have had an influential effect on participation.

The number of course attendees influenced the number of pilot study participants. However, the good recruitment rate resulted in a sample large enough to provide meaningful information as recommended for pilot studies [48, 49]. The majority of the sample was composed of nurses. The relatively large proportion of participants whose occupational affiliation was not specified (labeled as “others”) indicates an interest in the CIPE content, outside our initially targeted occupational groups.

Overall, participants were very satisfied with the CIPE intervention. The ratings in our study were comparable to that in the pilot study on the development of the ABC-SAT [46]. The high degree of satisfaction by participants using the ABC-SAT was also reflected in the oral feedback of the attendees (organization, content, didactic, learning experience); however, it can also be interpreted, albeit with caution, as a potential ceiling effect of the instrument [50]. Concerning reliability, internal consistency of the first two scales show only moderate/low Cronbach’s Alpha values. This may be explained with the very small item number of the subscales, being 3 and 2 items, respectively [46], since Cronbach’s Alpha tends to increase with higher item numbers [51, 52].

With respect to learning, 64.1% of the participants had a knowledge gain after the course. The knowledge test revealed a statistically significant improvement of participants’ knowledge upon participating in the course. However, this improvement was not observed when comparing the number of incorrect answers before and after the CIPE intervention, which may be due to a type II error. This can only be verified with a larger sample. Overall, one can assume that participants gained knowledge by attending the courses, despite a lower self-assessment of learning success in the ABC-SAT. The size of the effect is comparable to the results of a similar study by Brown et al. [53] that evaluated a trauma education program for people who work with children with severe emotional or psychiatric problems, and found an increase of 2 to 3 points in multiple choice knowledge tests.

The fact that we found no statistically significant correlation between satisfaction and learning could be due to the fact that a causal relationship is not assumed by Kirkpatrick [35] and evidence suggests that a causal relationship between Kirkpatrick’s levels 1 and 2 is unlikely [54]. The latter is supported by a study that examined this relationship in the context of management trainings which also found no correlation [55].

### Strengths and limitations

The strength of our pilot study is the formal representation of development, implementation, and evaluation of an educational intervention in the form of a CIPE that is one of the few of its kind. This created an evidence base that initially proves its feasibility, and provides insights regarding the reaction of and the effect on participants. Especially, the high degree of satisfaction can be understood as an indicator that the curriculum provides the right approach. Furthermore, the CIPE courses attracted interest from a wide variety of occupational groups within the health and social sectors, underlining the topic relevance with the preference for learning within interprofessional groups. The high level of fidelity of intervention delivery increased the comparability of the individual pilot courses, improving the meaningfulness of the evaluation outcomes [56]. On this basis, a further development and a possible permanent implementation of the CIPE intervention seems realistic, not least because of its relevance to current global events.

There are also several limitations. We only evaluated the first two levels and not the third (behavior) and fourth (results) levels of the Kirkpatrick model [35]. The assessment of whether the CIPE intervention has an effect on healthcare professional behavioral changes and organizational practice was not possible in a pilot study. This would have required further observations and analyses in the work areas of participants and exceeded available resources. However, the focus on the first two levels seems appropriate to meet the needs of local stakeholders [57] and corresponds to the procedure recommended by Kirkpatrick and Kirkpatrick [35].

Furthermore, internal and external validity are limited in our study. In the absence of a control group, the chosen research design does not allow to draw conclusions about a causal relationship between the CIPE intervention and investigated outcomes. In particular, a test effect due to the relatively short period between the knowledge test, before and after the CIPE intervention, might have biased its results [58, 59]. This applies in particular to the knowledge gain, which could be affected by a recall bias. The small sample size, although seeming acceptable for a pilot study [48], impairs statistical conclusion validity and might have produced false-positive results [60] which are not generalizable.

### Implications

The results of our pilot study provide indications of adaptation and development potential with regard to the curriculum and to the further evaluation of the CIPE intervention.

#### Curriculum

A prolongation of the CIPE courses would make it possible to integrate further content mentioned by participants (e.g. legal aspects) and to create more space for the exchange of experiences between attendees. Another possibility could be to extract content and make it available to attendees in advance as web-based information (e.g. introduction and background; literature recommendations) as proposed by Berggren et al. [61]. This would most likely save time in modules 1 and 2 of our CIPE intervention. At the didactic level, in order to increase the degree of interaction and to maintain effective learning, consideration should be given to (1) the implementation of further active learning strategies [62] to keep attendees active involvement as high as possible, to (2) the inclusion of more time for informal learning [63] (e.g. in the form of longer breaks), and (3) whether role-plays should be used or offered according to group dynamics.

Additional advertising with professional associations or educational institutions, outside the healthcare system, could positively impact the number of attendees. To this point, regular implementation could also contribute awareness of the CIPE intervention. To attract other professions, an extended needs assessment could be carried out in specific target groups [64].

#### Evaluation

There is a need to further evaluate the CIPE intervention. Future studies should be conducted with a larger sample, and ideally with a control group to improve internal and external validity. In addition, investigation into the extent to which the intervention affects the behavior of participants and ultimately the practice (3rd and 4th level of the Kirkpatrick model) in the long-term would be required. The characteristics of the sample in terms of job affiliation, subject area, or department of professional activity need to be considered in a more differentiated way, as they may involve different prior knowledge and skills which, like professional socialization [65], could influence learning.

## Conclusions

This pilot evaluation study confirmed the feasibility of a new evidence-based CIPE intervention for healthcare providers on the topic of PTSD within the context of flight and migration. Participants of the pilot courses showed high levels of satisfaction and a positive learning effect. On the basis of these results, the intervention can now be refined and should be evaluated in a larger scale study.

## Supplementary information

**Additional file 1.** Knowledge test for the continuing interprofessional education intervention entitled “posttraumatic stress disorder symptoms in patients with flight and migration history”. This document shows a translated version of the self-developed knowledge test used in the pilot evaluation study.

**Additional file 2.** Detailed content description of the final version of the curriculum for the CIPE intervention. This document provides a detailed description of the content of the final version of the curriculum and the pocket card.

## Data Availability

The datasets used and/or analyzed during the current study are available from the corresponding author on reasonable request.

## Abbreviations

CIPE: Continuing interprofessional education
CVI: Content Validity Index
I-CVI: Content Validity Index on item level
IQR: Interquartile range
PTSD: Posttraumatic stress disorder
S-CVI: Content Validity Index on scale level
SD: Standard deviation
