# Supplementary material for: Supporting migrants and refugees with posttraumatic stress disorder: development, pilot implementation, and pilot evaluation of a continuing interprofessional education for healthcare providers
Source: BMC Med Educ. 2020 Sep 16;20:311. doi: 10.1186/s12909-020-02220-3 (PMC7493357; doi:10.1186/s12909-020-02220-3)
Supplement: Supplementary file 1 — Additional file 1. Knowledge test for the continuing interprofessional education intervention entitled “posttraumatic stress disorder symptoms in patients with flight and migration history”. This document shows a translated version of the self-developed knowledge test used in the pilot evaluation study. [file 12909_2020_2220_MOESM1_ESM.pdf]

Knowledge test for the continuing interprofessional education intervention entitled "posttraumatic stress disorder symptoms in patients with flight and migration history"

Note: The knowledge test was translated into English and is displayed in a different formatting than the original Version.

(Correct answers are labeled with an asterisk [*])

1. What is posttraumatic stress disorder (PTSD)?
   (Multiple answers possible)
   a. A mental illness*
   b. A symptom of a disease
   c. A pathological reaction that occurs in every human being after a stressful event
   d. An imbalance of neurotransmitters in the brain
2. What are risk factors for the development of PTSD?
   (Multiple answers possible)
   a. Long duration of a traumatic event*
   b. Other mental illnesses*
   c. Migration
   d. Culture
3. Which factors protect against the development of PTSD?
   (Multiple answers possible)
   a. Stable and supportive relationships*
   b. High age
   c. High level of education*
   d. Origin or cultural background
4. What health care services can asylum seekers utilize in Germany?
   (Multiple answers possible)
   a. All
   b. Emergency treatments only*
   c. Rehabilitation only
   d. None
5. What can be the cause of PTSD?
   (Multiple answers possible)
   a. An inpatient stay in hospital*
   b. War experiences*
   c. Witnessing an act of violence*
   d. Hear / Watch reports from victims of a disaster*
6. Which of the following statements are true?
   (Multiple answers possible)
   a. Depending on a person's culture of origin, PTSD manifests itself differently*
   b. The majority of migrants in Germany apply for asylum
   c. A PTSD always develops directly after a trauma
   d. A trauma can cause different mental illnesses*

7. What can be symptoms of PTSD?
   (Multiple answers possible)
   a. Avoidance of certain situations/places/stimulus*
   b. Hallucinations
   c. Dissociations*
   d. Anxieties*
8. How can PTSD be treated?
   (Multiple answers possible)
   a. Psychotherapy*
   b. There is no specific treatment
   c. Psychotropic drugs as monotherapy
   d. Adjuvant ergotherapy or art therapy*
9. What other health problems can arise in connection with PTSD?
   (Multiple answers possible)
   a. None
   b. Pain*
   c. Depressions*
   d. Movement disorders
