# Supplementary material for: Supporting migrants and refugees with posttraumatic stress disorder: development, pilot implementation, and pilot evaluation of a continuing interprofessional education for healthcare providers
Source: BMC Med Educ. 2020 Sep 16;20:311. doi: 10.1186/s12909-020-02220-3 (PMC7493357; doi:10.1186/s12909-020-02220-3)
Supplement: Supplementary file 2 — Additional file 2. Detailed content description of the final version of the curriculum for the CIPE intervention. This document provides a detailed description of the content of the final version of the curriculum and the pocket card. [file 12909_2020_2220_MOESM2_ESM.pdf]

Detailed content description of the final version of the curriculum for the CIPE intervention

Module	Topic<br>Specific learning objectives<br>• Brief description of the content	Educational methods and materials	Duration (minutes)
1	Formal introduction<br>Attendees are informed about the procedure and the framework conditions of the continuing education<br>• Welcome<br>• Introduction of educators<br>• General information on the course	-	10
2	Thematic introduction<br>Attendees accommodate to the topic of the continuing education<br>Creation of group dynamics for constructive interaction<br>• Introduction of Attendees<br>• Moderated exchange about professional experiences on the topics trauma / traumatic experience and PTSD	discussion, experience exchange	20-30
3	Background information on trauma, PTSD & flight/migration<br>Attendees know about the disease PTSD and its causes, symptoms, diagnostics and therapy<br>Attendees are informed about the current status and developments on migration<br>Attendees gain insights into the connection between migration / flight and PTSD<br>• Disambiguation<br>• Pathophysiology<br>• Epidemiologic data<br>• Case example (Videos) : Report of refugee about the traumatizing flight from Africa to Europe and the experience of symptoms of PTSD	lectures, videos	50-60
Break 1			30
4	Handling symptoms of PTSD<br>Attendees are aware of PTSD in refugees<br>Attendees are familiar with interventions in dealing with the symptoms of PTSD in refugees<br>Attendees know possibilities of self-protection<br>• Possible practical applications of handling symptoms of PTSD in refugees/migrants and possible strategies of self-care for healthcare providers caring for traumatized patients.	lectures, brochure (pocket card), video	45-60

**Module**	<u>**Topic**</u><br>**Specific learning objectives**<br>• **Brief description of the content**	**Educational methods and materials**	**Duration (minutes)**
	• Video of an interview with an affected person (focus on experiencing hospital stays and needs and desires)<br>• The pocket card summarizes the main contents of this module and presents them in the form of recommendations with interventions for (a) dealing with people with symptoms of PTSD and (b) self-care of healthcare providers caring for their traumatized patients.<br>Main themes:<br>a) -What can be done: Promoting self-care; stress reduction; creation of a familiar and safe environment; promote integration; screen for further symptoms; Accept any emotions of the patient; suggestion of a psychiatric consultation<br>-What should be avoided: No activation of the trauma; do not address negative experiences; do not demand a description of the events; no trauma processing; no unannounced touch<br>b) -What can be done: Respect your own limits; demand further education / training; talk about it; use offers; examples for self-care interventions<br>-What should be avoided: No additional loads; do not ignore conflicts; do not ignore stress; do not ignore physical symptoms; renounce drugs/alcohol as compensation		
5	<u>Group work on handling symptoms of PTSD in refugees</u><br>Attendees internalize contents of the FB on the basis of the processing of a case vignette taking into account interprofessional cooperation<br>Attendees use tips and interventions presented in Modules 3 and 4<br>Attendees work out instructions for the case described in the vignette and present it afterwards<br>• Attendees can join one of three groups, in each of which one question on a case vignette is elaborated<br>• Presentation of the developed recommendations	self-directed learning, group work, experience exchange, presentation	45
Break 2			15
6	<u>Outlook on further aspects in the health care of refugees/migrants</u><br>Attendees know about further aspects and s in the care of people with migration / flight experience<br>• Regulations & barriers to health care services utilization<br>• Cultural aspects of illness perception Family<br>• Communication & Language	lecture	30

Module	<u>Topic</u><br>**Specific learning objectives**<br>• **Brief description of the content**	Educational methods and materials	Duration (minutes)
	• Possibilities to bridge language and communication barriers		
7	<u>Summary of key issues of the continuing education</u><br>Attendees know the central contents/statements of the course<br>• Joint elaboration of Take-Home-Messages<br>• Document with further information (handout) (for example recommended readings, addresses of public institutions)<br>• Questions	experience exchange, discussion;<br>handout	15-20
8	<u>Closing</u><br>• Feedback of attendees and training staff<br>• End/Farewell	-	15-20
